# *In Vivo* Antiplasmodial Activity of Two Sahelian Plant Extracts on* Plasmodium berghei* ANKA Infected NMRI Mice 

**DOI:** 10.1155/2018/6859632

**Published:** 2018-05-24

**Authors:** Léa Nadège Bonkian, R. Serge Yerbanga, Benjamin Koama, Aboubakar Soma, Mamoudou Cisse, Innocent Valea, Halidou Tinto, Jean Bosco Ouedraogo, T. Robert Guigemde, Maminata Traore/Coulibaly

**Affiliations:** ^1^Centre MURAZ, 01 BP 390 Bobo-Dioulasso 01, Burkina Faso; ^2^Institut de Recherche en Sciences de la Santé/Direction Régionale de l'Ouest, BP: 545 Bobo-Dioulasso, Burkina Faso; ^3^Institut des Sciences et Techniques, INSTech, BP: 2779 Bobo-Dioulasso, Burkina Faso; ^4^Université Nazi BONI, Bobo-Dioulasso, 01 BP: 1091 Bobo-Dioulasso 01, Burkina Faso; ^5^Centre National de Recherche et de Formation sur le Paludisme, 01 BP: 2208, Ouagadougou 01, Burkina Faso; ^6^Clinical Research Unit of Nanoro, 11 BP: 218 Ouagadougou CMS 11, Burkina Faso

## Abstract

Up to now, the control of malaria remains a challenge. The World Health Organization (WHO) recommends the use of artemisinin-based combination therapies (ACTs) for uncomplicated malaria treatment. Despite this guideline, many people in Burkina Faso use herbal medicine as primary treatment against malaria. The aim of this study was to assess the* in vivo* activity of* Guiera senegalensis* J. F. Gmel and* Bauhinia rufescens* Lam. leaves extracts against* Plasmodium berghei* ANKA. A four-day treatment of leaves decoction of each plant was administrated orally to 7 groups of six NMRI (Naval Medical Research Institute) mice infected with* Plasmodium berghei* ANKA strain. The control group received distilled water as treatment while the treated groups each received daily 100, 250, and 500 mg extract/kg body weight. Thin blood smears were performed on day five and the percentage of reduction of parasitaemia was determined compared to the control. The percentages of reduction of the parasitaemia at the doses of 100, 250, and 500 mg extract/kg body weight were, respectively, 57.5%, 35.9%, and 44.9% for* Guiera senegalensis* and 50.6%, 22.2%, and 25.7% for* Bauhinia rufescens. *Our findings on antiplasmodial activity of these two plants justify the traditional use by local populations against malaria. Thus, the isolation of the active compounds from these two plants is suggested for possible antimalarial candidate drugs.

## 1. Introduction

The control of malaria constitutes a challenge for endemic countries. In 2015, the number of worldwide cases of malaria was estimated at 212 million with populations in developing countries at higher risk [1]. In 2014, 7,814,634 cases of uncomplicated malaria, 463,774 cases of severe malaria, and 5632 deaths were registered in Burkina Faso [2]. The World Health Organization recommended the use of artemisinin-based combinations therapies (ACTs) for the first-line treatment of uncomplicated malaria [3]. Despite this guideline, about 80% of people in Burkina Faso continue to use plant recipes to treat themselves particularly in rural areas (Yelkouni, personal communication). Surveys have been conducted and plants have been selected to assess their antimalarial properties through* in vitro* and* in vivo* biological assays. The assessment of the* in vitro* antiplasmodial activity of* Cassia sieberiana* leaves extracts showed a very good activity (100% of* Plasmodium* elimination) [4]. The stem extract (DCM/MeOH) of* Momordica balsamina *showed a promising activity (IC50 = 5.3 *μ*g/ml) [5]. The* in vitro* decoction of* Azadirachta indica *A. Juss showed a high* in vitro* activity (IC50 = 4.69 ± 1.1 *μ*g/ml). Even if the efficacy of some plants has been demonstrated in suitable antimalarial models, further studies are needed to evaluate the antimalarial properties of a greater number of plants. An ethnobotanical survey conducted in Sahel region of Burkina Faso reported that about 40 plant species were used by traditional healers in recipes for malaria treatment [6]. The aim of the present study was to assess the* in vivo *activity of* Guiera senegalensis* J. F. Gmel and* Bauhinia rufescens* Lam. leaves extracts against* Plasmodium berghei* ANKA.

## 2. Materials and Methods

The study was conducted in Burkina Faso in October 2017. It was an experimental study testing the* in vivo* activity of two plant extracts on mice from the Naval Medical Research Institute (NMRI) infested with* Plasmodium berghei* ANKA strain.

### 2.1. The Plant Material

Leaves of two plant species were collected for the study:* Guiera senegalensis* J. F. Gmel, a shrub about 3 m high belonging to Combretaceae family, and* Bauhinia rufescens* Lam., a small tree 1–8 m high belonging to Fabaceae family.

The plant parts were harvested in Gorom-Gorom area in the Sahel Region of Burkina Faso in June 2017. The two species were identified by Dipama Pascal at IRSS (Institut de Recherche en Sciences de la Santé), and voucher specimens GsL2017 and BrL2017, respectively, for* Guiera senegalensis* leaves and* Bauhinia rufescens* leaves have been deposited in the Unit of Pharmacognosy at IRSS Bobo-Dioulasso. The plant materials harvested were dried in the shade at ambient temperature (about 30–35°C) for 7 days and then milled. The powder was stored in bags at ambient temperature in a dried area until the extraction.

Decoctions of each of the leaves materials from the plants were prepared by boiling 50 g of the powder in 500 mL of distilled water over a period of 30 minutes. The extracts were filtered, dried by lyophilisation, and stored at 4°C.

### 2.2. The Biological Material

- Forty-two (42) female NMRI mice, 7 to 10 weeks of age, weighing between 24 and 30 g, were used for the assessment. The mice were obtained from CIRDES (Centre International pour la Recherche-Développement pour l'Elevage en Zone Sub-Humide) and sent to the “Institut de Recherche en Sciences de la Santé” (IRSS/DRO) where the assessment was performed. Before starting the experiment, the mice were divided into 7 groups of 6 mice, acclimatized at 25°C ± 2°C, with 12 h photoperiod per day, and fed with standard food and water for 2 days before treatment. Each mouse was marked to permit their individual identification.
- The parasites used were chloroquine sensitive* Plasmodium berghei* ANKA strain (MRA-311, MR4, ATCC, Manassas, Virginia) stored at −80°C.

### 2.3. Preparation of Treatment Doses

The mass of the dried extract needed for each dose preparation was calculated based on the average of each group's weight. The doses were prepared by diluting the dried extract corresponding to the weight of the mouse in 200 *µ*L of distilled water. For example, 100 mg/Kg dose for mouse of 25 g was obtained by diluting 2.5 mg of dried extract in 200 *µ*l of distilled water. Then, mice were treated by oral route with 200 *µ*l of this solution.

### 2.4. *In Vivo* Tests

The* in vivo* antiplasmodial activity was performed according to the 4-day suppressive test [7]. The mice infection was done by injecting* Plasmodium berghei* ANKA infected blood strain to 4 naive mice via intraperitoneal route. Four days after the infection, the parasitemia of these mice (donor mice) was determined and their blood was collected and suspended in a Phosphate Buffered Saline (PBS) solution. 10^7^* Plasmodium berghei* infected erythrocytes of this blood were inoculated intraperitoneally to the 7 groups of mice. Two (2) hours after the inoculation, a treatment is administrated orally to each mouse. The first three groups of mice received, respectively, 100, 250, and 500 mg of* Guiera senegalensis* extract per kg body weight every day; and the second 3 groups received the same doses of* Bauhinia rufescens* leaves extract and the last group which is the control received everyday distilled water. Each dose was administrated once daily during a period of 4 days. On the fifth day, thin blood smears were performed from a drop of blood taken from tail snip of each mouse and the slides were fixed with methanol and stained with 10% Giemsa solution. The parasitemia was determined with a microscope by counting the number of parasitized red blood cells (RBCs) on 3 randomly selected fields on each slide. The percentage of parasitemia is

(1) $$\begin{array}{c} \%\text{ Parasitemia}=\frac{\left(\text{Number of infected RBCs}\right)}{\text{Total Number of RBCs}}\times 100 \end{array}$$

The extract activity is determined by calculating the percentage of reduction of parasitemia:

(2) $$\begin{array}{c} \%\;reduction=\frac{\left(\%\text{ parasitemia of Negative control }-\%\text{ parasitemia of Treated group}\right)}{\%\text{ parasitemia of Negative control}}\times 100 \end{array}$$

### 2.5. Data Analysis

The data were entered and analyzed with Excel 2013 software and Epi Info 6.04. The results were expressed as mean of parasitemia ± standard deviation. The Student test was used to compare the results. The data were analyzed at 95% confidence interval and a value of *p* < 0.05 was considered as significant. The activity is classified as follows:

- Moderate when the percentage of reduction of parasitemia is equal to or greater than 50% at the dose of 500 mg extract/kg body weight.
- Good when the percentage of reduction of parasitemia is equal to or greater than 50% at the dose of 250 mg extract/kg body weight.
- Very good when the percentage of reduction of parasitemia is equal to or greater than 50% at the dose of 100 mg extract/kg body weight [8].

### 2.6. Ethical Considerations

This study protocol has been approved by the institutional ethic review board of “Centre MURAZ” (ref.: A 005-2014/CE-CM). The animals were used in accordance with the Directive 86/609/EEC.

## 3. Results

None of the experimental mice died during the 4-day test. The* in vivo* activity was determined by the reduction of the parasitemia of the dose of each decoction with reference to the control. The results of the tests were summarized in Tables 1 and 2.

The results indicated that the decoction of* Guiera senegalensis* leaves exhibited the highest inhibition of the parasitemia. The comparison of the test groups and the control group indicated that the decoction reduced significantly the level of the parasitemia at the doses of 100 mg/kg body weight (*p* = 0) and 250 mg/kg body weight (*p* = 0.000002) but not at the dose of 500 mg/kg body weight (*p* = 0.13). The* Guiera senegalensis* leaves extract indicated a significant difference of reduction of parasitemia between the doses 100 and 250 mg/kg body weight (*p* = 0.0005) with the highest level of reduction of the parasitemia at the dose of 100 mg/kg body weight.

The comparison of the test groups and the control group indicated that the decoction reduced significantly the level of the parasitemia at the dose of 250 mg/kg body weight (*p* = 0.00002) but not at the doses of 100 mg/kg body weight (*p* = 0.09) and 500 mg/kg body weight (*p* = 0.28). The activity at the dose of 250 mg/kg body weight was moderate.

## 4. Discussion

The plants used in the present study,* Guiera senegalensis* and* Bauhinia rufescens*, were chosen after an ethnobotanical study realized among the traditional healers in the Sahel Region of Burkina Faso [6]. The plant parts were harvested by a traditional healer in the region of Gorom-Gorom, a village located in the Sahel Region of Burkina Faso. This region, characterized by the dryness of its climate, is located in the northern region of Burkina Faso where transmission of malaria occurs in the short rainy season (3-4 months/year) leading to severe malaria among children [9].* Plasmodium berghei* ANKA is responsible for rodents' cerebral malaria which has some similarities with human cerebral malaria caused by* Plasmodium falciparum* [10]. A decoction of each plant was prepared in accordance with the mode of preparation used by the healer. A positive control was not used in this study because the aim of the study was to test the activities of the extracts on the parasite development.

The results of this study showed a good activity of* Guiera senegalensis* leaves decoction at the dose of 100 mg/kg body weight justifying the use of this plant in recipes used against malaria [6, 11]. The reduction of the parasitemia at the dose of 100 mg/kg of body weight was higher than at the dose of 250 mg/kg body weight. The pharmacokinetics of the extracts could be explained by the saturation of the plasma protein binding sites and also the possibility of toxicity at the highest doses. The activity of* Guiera senegalensis* could be explained by the phytochemistry of the plant which has already been documented [12]. Previous study showed that* Guiera senegalensis *leaves extract contains tannins, flavonoids, naphthopyrans, naphtyl butenone, and alkaloids [11, 13]. Fiot et al. had shown that the alkaloids responsible for the* in vitro* antiplasmodial activity seen in* Guiera senegalensis* leaves are harman and tetrahydroharman [11]. The decreasing of the activity with the increasing of the dose could also be explained by a technical problem due to the high concentration of the extract causing a problem of solubility.

The antiplasmodial activity of* Bauhinia rufescens* leaves extract was moderate at the dose of 250 mg/kg body weight but there was no significant difference with the mean of parasitemia of the control and the dose of 100 and 500 mg/kg body weight. Garbi et al.'s study showed that the leaves extract of* Bauhinia rufescens* contains flavonoids, tannins, triterpenes, saponins, and alkaloids [14]. The presence of the alkaloids in the extract could then explain the moderate antiplasmodial activity observed. However, if a previous study revealed that* Bauhinia rufescens* is used in some recipes against malaria, this* in vitro* study demonstrated that there is no significant antiplasmodial activity in any extract of this plant [15]. But to the best of our knowledge, a similar study was not found, thus making this the first one.

## 5. Conclusion

The study showed that, out of the two extracts,* Guiera senegalensis* J. F. Gmel leaves extract gave higher activity compared to* Bauhinia rufescens* Lam., making it a good candidate for antiplasmodial drugs. But further studies on its various compounds and potential activities in response to* Plasmodium berghei* ANKA must be done. Though* Bauhinia rufescens* Lam. showed no significance activity, when coupled with* Guiera senegalensis*, it may enhance its moderate activity or cause a synergic effect that may give a higher activity more than* Guiera senegalensis *alone. Thus, such combination must be investigated.

## Figures and Tables

**Table 1:** Antiplasmodial activity of *Guiera senegalensis* leaves extract.

Doses (mg/kg)	mean of parasitemia	percentage of reduction
Distilled water	33.4 ± 2,1	0
100	14.2 ± 2,3	57.5
250	21.4 ± 3,2	35.9
500	18.4 ± 11,1	44.9

**Table 2:** Antiplasmodial activity of *Bauhinia rufescens* leaves extract.

Doses (mg/kg)	mean of parasitemia	percentage of reduction
Distilled water	33.4 ± 2,1	0
100	16.5 ± 9,2	50.6
250	26 ± 1,9	22.2
500	24.8 ± 14,3	25.7

## Data Availability

All the data are available in the manuscript.
